# Connecting Medical Personnel to Dentists via Teledentistry in a Children's Hospital System: A Pilot Study

**DOI:** 10.3389/froh.2021.769988

**Published:** 2021-12-09

**Authors:** Kimberly J. Hammersmith, Macaire C. Thiel, Matthew J. Messina, Paul S. Casamassimo, Janice A. Townsend

**Affiliations:** ^1^Nationwide Children's Hospital, Columbus, OH, United States; ^2^The Ohio State University College of Dentistry, Columbus, OH, United States

**Keywords:** teledentistry, emergency department, urgent care, dentistry, dental trauma

## Abstract

Investigators evaluated feasibility, acceptability, and sustainability of a teledentistry pilot program within a children's hospital network between March, 2018, and April, 2019. The program connected dentists to medical personnel and patients being treated in urgent care clinics, a primary care clinic, and a freestanding emergency department via synchronous video consultation. Three separate but parallel questionnaires evaluated caregiver, medical personnel, and dentist perspectives on the experience. Utilization of teledentistry was very low (2%, 14/826 opportunities), but attitudes regarding this service were largely positive among all groups involved and across all survey domains. Uptake of new technology has barriers but teledentistry may be an acceptable service, especially in the case of dental trauma.

## Introduction

Over the last two decades, increasing numbers of pediatric patients have sought care at emergency departments (ED), urgent care (UC) facilities, and medical clinics with oral complaints including toothaches, dental trauma, and other concerns [1]. Medical providers have little oral health training and education [2]. Treatment for dental problems by medical providers is limited and may be managed by ineffective prescriptions for opioids or antibiotics. Patients receiving oral health care in an ED are more than 7 times more likely to receive an opioid prescription than patients treated in a dental office [3]. About 65% of patients receive antibiotics for their dental problem from medical providers when the indicated treatment for their condition is a procedure performed by a dentist [4]. Challenges related to low socioeconomic status, inadequate access to dentists particularly in rural areas, and low oral health literacy contribute to patients seeking dental care in ED or other medical facilities [5].

Inappropriate care seeking at medical facilities for oral concerns can be addressed by different interventions. More than half of patients arrive during business hours when dental offices are normally open [6]. Programs diverting patients from ED and UC centers and connecting them with dentists are one option [7]. Telehealth is another possible adjunct to provide guidance for patients physically located in medical settings while simultaneously exposing medical personnel to best practices from a dentist.

Telemedicine, connecting medical providers with other medical providers, has been feasible and acceptable for efforts in underserved areas [8] and improves the quality of care for patients with chronic conditions [9–12]. Additionally, telemedicine consultations with specialists produces better outcomes during acute or emergency care [13–16]. A 2017 study showed that mental health and primary care were the most common forms of telemedicine [17].

Barriers to successful implementation of telemedicine programs include state medical and dental licensing restrictions, inadequate reimbursement, and prohibitive legislative policy [18, 19]. Suboptimal financial reimbursement may hinder widespread adoption and sustainability of telemedicine [8]. All fifty states provide some Medicaid reimbursement for telehealth services, but differ dramatically in defining and regulating telehealth, so reimbursement may not meet expense [20]. Live, synchronous video visits are reimbursed the highest; asynchronous modalities often have restrictions. Some states put restrictions on eligible providers, eligible facilities, or require specific informed consent requirements [20].

Teledentistry is the use of teleconferencing software and cameras that allow a dentist to evaluate a patient remotely [21]. Cameras and software allow a live feed conference, synchronous consultation, or saving of images to be evaluated later, called asynchronous consultation [21]. Teledentistry was utilized by the Department of Defense in the 1990s in a pilot project aimed at patient care, continuing education, and dentist-laboratory communication [22]. Currently sixteen state laws include language specific to teledentistry [20].

Many examples of telemedicine connecting medical professionals to other medical professionals exist, but no studies have examined use of teleconsultation between dental and medical professionals. Only one study has suggested it as a means to improve interdisciplinary communication [23]. In late 2018, Ohio passed legislation that allows remote, synchronous consultations by a dentist to be recognized and billed similarly to an in-person encounter [24]. The aim of this study was to evaluate the feasibility, acceptability, and sustainability of a pilot teledentistry program in a children's hospital network in Ohio.

## Materials and Methods

Nationwide Children's Hospital in Columbus, OH, USA, launched a pilot teledentistry program in March 2018, connecting a free-standing, suburban ED with its dental department. The ED was equipped with a cart with a laptop, external speaker, webcam, and intraoral camera. Two dentists (MT and KH) trained the staff on teledentistry protocol and how to use equipment. When a patient presented with an oral- or dental-related chief complaint, the ED provider was to order a consultation with the on-call dental resident who would be paged, and then connect to the ED via a secure, synchronous platform HealthChat® (Miami, FL, USA). The dentist could then communicate directly with patient and family as well as ED staff regarding care.

Due to low utilization of teledentistry by the ED in the first 8.5 months, three UC sites and one primary care site with higher volumes of oral-related diagnostic codes were added to the same protocol to increase encounters for a total of five sites. We focused our data analysis once all five sites were included, for a period of 5 months.

This study to evaluate the pilot program was approved as exempt from the Nationwide Children's Hospital Institutional Review Board. Data from electronic medical records for all five sites was gathered including patient demographics, encounter information, and ICD-10 codes (Table 1) involving oral- or dental-related chief complaints.

**Table 1:** Frequency of dental diagnosis codes at five medical sites during pilot period.

		**Pilot period encounters**, ***N*** **=** **826**		**Teledentistry encounters**, ***N*** **=** **14**	
**ICD-10 diagnosis codes**	**Description**	***N***	**Percent %**	***N***	**Percent** **%**
K00.1, K09.0, K09.8, K12.0, K12.1, K12.30, K13.0, K13.21, K13.4, K13.70, K13.79	Lesions, cysts, pathology	266	32	0	0
K04.7, K12.2, L03.211, L03.213	Cellulitis or infections	151	18	3	21
K00.2, K02.9, K08.89, K08.9	Caries and tooth disorders	153	19	3	21
K00.6, K00.7	Teething and eruption	134	16	0	0
K05.00, K05.5, K05.6, K05.10, K05.219, K06.1, K06.8, K06.9	Gingivitis and periodontal disease	73	9	1	7
K03.81, K08.109, S02.5XXA, S02.5XXB, S03.2XXA, S03.2XXD	Trauma	43	5	7	50
K11.1, K11.20, K11.21, K11.6, K11.7, K11.8	Salivary glands	31	4	0	0
	Total	851*	100	14	100

* *851 codes noted, with 826 individual patients, as some patients received multiple diagnostic codes during their encounters*.

Three separate surveys were developed with duplicate or similar questions when possible to evaluate the teledentistry program as to its acceptability, feasibility, and sustainability. Surveys for the caregivers, the medical personnel initiating the teledentistry consultation, and the consulting dentist were adapted from models in the literature [25, 26]. Survey domains included: connection and equipment, comfort, competence of provider, teledentistry as substitute for in-person examination, access, workflow and efficiency, and preference. Following demographic questions gauging age, role, and experience with telemedicine technology, medical personnel answered 15, 5-point Likert scale type questions (14 such questions on the dentist survey). The survey intended for caregivers consisted of 15, 5-point Likert scale type questions and 4 questions had three answer choices.

The caregiver accompanying each child received a survey directly following a teledentistry encounter, while still in the ED. Exclusion criteria were caregivers who did not consent or were non-English speaking. Surveys were administered via paper for caregivers and REDCap [27], a secured web application, for dentists and medical personnel, who completed the surveys electronically soon after the encounter. Data was assessed using descriptive statistics only. Caregivers received a gift card as an incentive for completing the survey.

## Results

During the pilot period of 5 months across the five sites, 826 patients presented with an oral or dental complaint with average of 165 patients a month, though some would have been excluded from participating in teledentistry due to language barriers. A dentist was only consulted via teledentistry 14 times or <2% of possible encounters. During the pilot, the chief complaint of dental trauma comprised 5% of total visits but 50% of the teledentistry encounters (Table 1). A chief complaint of a lesion, cyst, or pathology occurred in 266 (31%) of dental-related encounters but resulted in zero teledentistry consults.

**Figure 1:**
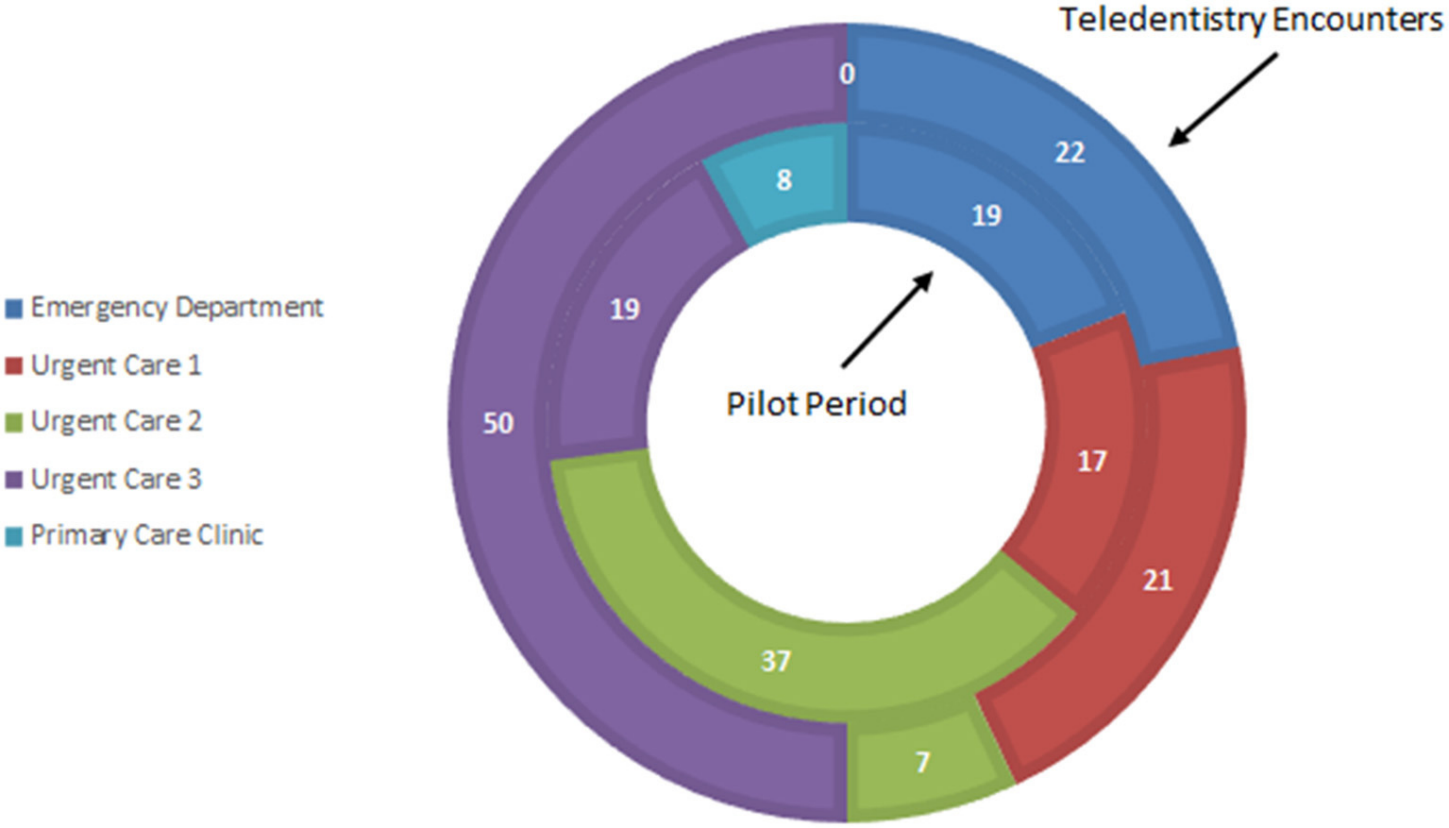
Location of patients (%) with oral or dental chief complaint.

During the pilot, the majority of teledentistry consults originated in one of the UC sites (50%), although patients with dental diagnosis codes arrived at all five sites (Figure 1). There were zero encounters from the primary care site. The disposition of all patients with a dental diagnosis code was 98% discharged, and 2% transferred to the main ED. Of the 14 teledentistry patients, 79% were discharged, and 21% were transferred.

Ten encounters included survey data from all three involved parties: patient/family (*n* = 14), medical personnel (*n* = 14 surveys, from 11 unique respondents), and dentist (*n* = 14 surveys, from 7 unique respondents). Medical personnel surveyed included 5 registered nurses and 6 physicians with some repeated, or non-unique, users. The majority were 31–50 years old, 7 (55%) were using teledentistry for the first time, and 11 (92%) had no previous experience using telemedicine technology for other services.

Table 2 displays the questions asked across all domains in each survey, as well as the overlap of questions across survey respondents. All medical personnel felt videoconferencing software and maneuvering the intraoral camera were easy. Directing and instructing medical personnel to use the intraoral camera was considered easy by most dentists, but in 3 encounters (21%), the dentist found it difficult. Comments indicated the need for better training of medical personnel on use of the intraoral camera (Table 3). All dentists but one (93%, *n* = 13) felt the software was easy to use and just over half (58%, *n* = 8) of dentists felt they could hear and visually assess the patient's area of concern as well as if they were present. The majority of caregivers (92%, *n* = 11) felt the dentist could hear and see their child's problem as well as if in the room.

**Table 2:** Selection of teledentistry survey questions by domain.

**Patient**	**Medical personnel**	**Dentist**
**Connection and equipment**		

N/A	Using the videoconferencing software (Healthchat) was:	Using the videoconferencing software (Healthchat) was:
	VE: 33%, E: 42%, N: 25%, D: 0%, VD: 0%	VE: 43%, E: 50%, N: 0%, D: 7%, VD: 0%
N/A	Maneuvering the intraoral camera for live video was:	Directing and instructing medical staff to use the intraoral camera was:
	VE: 42%, E: 50%, N: 8%, D: 0%, VD: 0%	VE: 7%, E: 57%, N: 14%, D: 21%, VD: 0%
N/A	The instructions for connecting to Healthchat were clear and accurate	The instructions for connecting and conducting a consult via Healthchat were clear and accurate
	SA: 50%, A: 42%, N: 8%, D: 0%, SD: 0%	SA: 43%, A: 57%, N: 0%, D: 0%, SD: 0%
I think the dentist could hear and see my child's problem as well as if the dentist were in the room	N/A	I could hear and visually assess the patient's area of concern as well as if I were there in person
SA: 58%, A: 33%, N: 8%, D: 0%, SD: 0%		SA: 29%, A: 29%, N: 29%, D: 14%, SD: 0%

**Comfort**		

I was comfortable with the teledentistry process	I was comfortable with the teledentistry process	I was comfortable with the teledentistry process
SA: 75%, A: 25%, N: 0%, D: 0%, SD: 0%	SA: 42%, A: 50%, N: 8%, D: 0%, SD: 0%	SA: 50%, A: 43%, N: 0%, D: 7%, SD: 0%
Someone explained to me that we were going to use teledentistry so that I knew what to expect	N/A	N/A
Yes: 100%, No: 0%		
I feel that my personal information was protected during the interaction with the dentist	N/A	N/A
SA: 83%, A: 8%, N: 8%, D: 0%, SD: 0%		

**Competence of provider**		

The dentist understood my child's problem	I think the dentist understood the patient's problem	I was able to gather necessary information to make a recommendation or diagnosis I felt comfortable with, as I would with a non-teledentistry patient
SA: 75%, A: 17%, N: 8%, D: 0%, SD: 0%	SA: 83%, A: 17%, N: 0%, D: 0%, SD: 0%	SA: 50%, A: 43%, N: 0%, D: 7%, SD: 0%
The dentist responded to my concerns	N/A	N/A
SA: 67%, A: 25%, N: 8%, D: 0%, SD: 0%		
The dentist explained what's going on with my child's teeth or mouth	N/A	N/A
SA: 58%, A: 25%, N: 17%, D: 0%, SD: 0%		

**Teledentistry as substitute for in-person examination**		

My child was given the same recommendations as if the dentist were here in person	N/A	The patient was given the same recommendations as if I were there in person
SA: 66%, A: 17%, N: 17%, D: 0%, SD: 0%		SA: 71%, A: 29%, N: 0%, D: 0%, SD: 0%
N/A	I think still photos rather than video would have allowed the dentist to provide the same diagnosis	I think still photos rather than video would have allowed me to provide the same diagnosis
	SA: 8%, A: 25%, N: 50%, D: 8%, SD: 8%	SA: 7%, A: 50%, N: 21%, D: 21%, SD: 0%
N/A	N/A	I think the patient would have the same satisfaction if still photos rather than video were used
		SA: 0%, A: 0%, N: 14%, D: 71%, SD: 14%
The doctor here at this patient care site could have managed my child's needs fine without getting the dentist on the computer	N/A	The intraoral camera gave me a view of the problem equivalent to if I were there in person
SA: 8%, A: 17%, N: 50%, D: 17%, SD: 8%		SA: 14%, A: 64%, N: 7%, D: 14%, SD: 0%

**Access**		

I would like this patient care site to keep offering this service to patients	I would like this patient care site to keep offering this service to other patients	I would like this medical location to keep offering this teledentistry service to other patients
SA: 92%, A: 8%, N: 0%, D: 0%, SD: 0%	SA: 58%, A: 42%, N: 0%, D: 0%, SD: 0%	SA: 79%, A: 21%, N: 0%, D: 0%, SD: 0%
Had I not had access to the dentist on the computer today, I would not know what to do for my child's problem	Had the dentist not been available, the care of this patient would have been compromised	N/A
SA: 67%, A: 25%, N: 8%, D: 0%, SD: 0%	SA: 8%, A: 17%, N: 42%, D: 33%, SD: 0%	
I would recommend the teledentistry process to a friend in a situation similar to mine	I would recommend teledentistry to my colleagues	N/A
SA: 75%, A: 25%, N: 0%, D: 0%, SD: 0%	SA: 58%, A: 42%, N: 0%, D: 0%, SD: 0%	
My insurance should cover this service	I think that teledentistry improves the quality of services provided at this patient care site	N/A
Yes: 75%, Maybe: 25%, No: 0%	SA: 67%, A: 33%, N: 0%, D: 0%, SD: 0%	

**Workflow and efficiency**		

I am satisfied with how long I got to talk to the dentist on the computer	It takes more than one staff member to use the teledentistry equipment	I think that I would be able to treat more patients using teledentistry
SA: 67%, A: 33%, N: 0%, D: 0%, SD: 0%	SA: 17%, A: 25%, N: 8%, D: 50%, SD: 0%	SA: 79%, A: 21%, N: 0%, D: 0%, SD: 0%
I am satisfied with how long I waited to talk to the dentist	The teledentistry consult took an appropriate amount of time	N/A
SA: 75%, A: 25%, N: 0%, D: 0%, SD: 0%	SA: 50%, A: 25%, N: 17%, D: 8%, SD: 0%	
Talking with the dentist on the computer today was worth the time that I saved in not having to go to Nationwide Children's main emergency department downtown	Talking with the dentist on the computer was a good use of the patient's time	N/A
SA: 50%, A: 17%, N: 33%, D: 0%, SD: 0%	SA: 75%, A: 25%, N: 0%, D: 0%, SD: 0%	
N/A	This consult method fits well into the “process or flow” of this patient care site	N/A
	SA: 50%, A: 33%, N: 17%, D: 0%, SD: 0%	

**Preference**		

In this location, I would prefer:	N/A	I would prefer to consult patients similar to this patient via teledentistry rather than in person
Dentist in person: 33%, No preference: 58%, Dentist on computer: 8%		SA: 43%, A: 43%, N: 7%, D: 7%, SD: 0%
In this location, I would prefer:	N/A	N/A
Dentist on computer: 33%, No preference: 50%, No dentist available for today's visit: 17%		
In this location, I would prefer:	N/A	N/A
Dentist on computer: 33%, No preference: 50%, Transfer to ED downtown: 17%		

*VE, Very easy; E, Easy; N, Neutral; D, Difficult; VD, Very difficult; SA, Strongly agree; A, Agree; N, Neutral; D, Disagree; SD, Strongly disagree*.

**Table 3:** Comments from caregivers, medical personnel, and dentists.

**Caregiver**	**Medical personnel**	**Dentists**
• It was good and helpful for all the service • 100% Perfect • None, you did great! • It was really kind and helpful for the dentist to help my daughter and check out for her issue or helpful. Really appreciate for the kindness!	• For some reason the video from the wand with the covering was blurred. This may have been operator (me) error • it was a little time consuming using the Teledentistry software, but I suspect my efficiency and ease of use will improve with time • The ED staff was either intimidated by the technical process or just unwilling to help (I kept hearing 'I've never done it') so I had to take the time to read through the directions while patients were stacking up • The teledentistry for this patient was very beneficial. It was determined that she needed to be admitted for IV antibiotics. The family didn't have any transportation to the hospital. They took the COTA bus to our facility. We couldn't send them by public transportation to the hospital. I feel that with being able to speak with the dentist in real time helped the family understand the need for us to arrange a taxi to take them. The family remained calm even though it took a while to make the arrangements. I feel it was all due to the dentist explaining the complex situation to the family • The amount of time needed was excessive due to technical glitches in the software. Multiple events were created due to staff not being able to see the other person • We had some initial issues with the connection but once that was figured out it ran very well. The intraoral camera was hard to use at first but never used one before. Got easier as time went. This was a great service!! Parents were so appreciative of having the dental consult there and then. Thank you!!	• Some quick training of medical staff regarding how to retract the cheek, lip, or tongue when using the intraoral camera would be helpful • I think this was great - especially for those who live far and don't have easy access to NCH. It allows for assessment and triaging of true emergencies versus dental problems that can wait until the next day. This will help alleviate the worry parents have as well as freeing up resources in emergency rooms. As far as training - I think it would be beneficial to continue more training with how to maneuver the intraoral camera. This would include retracting the cheek when using the camera. • I think that teledentistry will work very well but the healthcare providers need to be trained in the use of the intraoral camera. I spent around 10 minutes trying to coach the physician to put the software on the correct camera so I could see anything at all. If providers are not tech savvy it may be more useful to have them describe or simply take a picture. • Camera issues, only able to see via intraoral camera, not chat/videoconferencing camera. Family seemed reassured and glad to have a consult with a dentist. • Having a training session on how to best retract the lips/tongue would be helpful for the physician and perhaps help him/her feel more comfortable when positioning the intraoral camera • Staff had a difficult time positioning the camera for me to get a diagnostic view. I believe with more practice/training it would be fine. Are medical staff able to see what they are showing the dentist on the intraoral camera? Perhaps they were not looking in the right place but that would be helpful for them. They kept asking 'can you see the teeth now?' But the camera would be showing the ceiling, up the pt's nose and other parts of the room! Patients seemed to appreciate being able to talk to me directly which is a benefit over still photos.

Regarding the comfort domain, most medical personnel (92%, *n* = 11) and dentists (93%, *n* = 13), as well as 100% (*n* = 12) of caregivers were comfortable with the process. All caregivers surveyed said that teledentistry was explained and they knew what to expect, and all but one (91%, *n* = 11) agreed their personal information was protected during the interaction with the dentist.

As far as provider competence, all medical personnel (*n* = 12) thought the dentist understood the patient's problem. Thirteen dentists (93%) felt able to gather necessary information to make a confident diagnosis or recommendation, as they would with a non-teledentistry patient. Overall, caregivers felt that the dentist understood their child's problem (92%, *n* = 11), and responded to their concerns (92%, *n* = 11) and the majority agreed the dentist explained their child's situation (83%, *n* = 10).

Regarding teledentistry as a substitute for in-person examination, some dentists (57%, *n* = 8) and medical personnel (33%, *n* = 4) agreed that still photographs would have allowed the dentist to make the same diagnosis as synchronous video. However, most dentists (85%, *n* = 12) disagreed that patient satisfaction would be the same if still photos were used rather than video. When asked if the intraoral camera view of the patient's problem were equivalent to an in-person assessment, only 2 (14%) dentists disagreed. Most caregivers (83%, *n* = 10) and all dentists (100%, *n* = 14) felt the patient received the same recommendations as if the dentist were present. When caregivers were asked if a medical provider could have managed alone, without contacting the dentist, 3 (25%) agreed, 6 (50%) were neutral, and 3 (25%) disagreed.

As far as access, all 12 medical personnel agreed teledentistry improves the quality of services provided at their site. If the dentist had not been available, 3 (25%) medical personnel thought their patient's care would have been compromised. All patients and medical personnel would recommend teledentistry in a similar situation. All patients, dentists, and medical personnel indicated they would like the medical site to keep offering teledentistry.

For workflow and efficiency, the majority (75%, *n* = 9) of medical personnel felt the teledentistry encounter took an appropriate amount of time, was a good use of patient's time (100%, *n* = 12), and that this consult method fits well into the site process or flow (83%, n=10). Five medical personnel (42%) felt more than one staff member was needed to use the teledentistry equipment. All 14 (100%) dentists thought they would be able to treat more patients using teledentistry. Caregivers were satisfied with time available to speak with the dentist (100%, *n* = 12) and wait time to connect (100%, *n* = 12). The majority (67%, *n* = 8) agreed that teledentistry was worth the time saved by not having to go to the main ED that has dental services.

Regarding preference, when caregivers were given the option between teledentistry and dentist in person, the majority (58%, *n* = 7) responded neutral, with one (8%) choosing teledentistry. Given the options of teledentistry or no dentist at all, six (50%) responded neutral, with four (33%) preferring teledentistry, and two (17%) no dentist at all. Given options of teledentistry or transferring to the main ED, six (50%) caregivers responded neutral, with four (33%) preferring teledentistry and two (17%) transfer to the main ED. As far as dentist preference, 12 dentists (86%) preferred teledentistry to in-person consultation.

## Discussion

This study is the first to describe how teledentistry can be used to facilitate consultation and communication between dentists and medical personnel and revealed barriers in connecting the two. The pilot project utilized live, hands-on trainings at each site, with regular assessments and updates via email. Medical personnel initially expressed interest and enthusiasm for the new technology, but despite coaching, reminders, incentives, and motivation, the pilot utilization of teledentistry technology was extremely low.

Comments from the medical personnel regarding intimidation by the technology and added workflows may surmise why utilization was low. In a busy medical setting, provider perception of a larger burden-over-benefit may have contributed to the low number of teledentistry encounters. Importantly, surveys showed that medical personnel's experiences were generally positive when teledentistry was used, and individual comments about the utility for specific patients support this sentiment further.

Additionally, medical providers may perceive they are competent in triaging and managing oral- or dental-related chief complaints in medical settings, resulting in the minimal uptake of teledentistry. Compared to common diagnoses of teething and caries, teledentistry was used disproportionately for trauma codes suggesting medical providers felt less comfortable or familiar with these presentations. As more teledentistry patients were transferred to the main campus ED than non-teledentistry patients (21 vs. 2%), we see that the technology was notably utilized for more severe cases.

Even though medical personnel may have had some issues with the intraoral camera and teledentistry software, the patients liked the technology and appreciated the opportunity to speak to a dental provider when a dentist would otherwise not be accessible. As most answers were positively answered by both dentists and medical personnel, the project appeared to be acceptable.

The few encounters limit our ability to report meaningful survey data and associations with variables such as demographics. We had originally intended to compare length of stay, disposition, and patient variables such as age, race, and insurance status for teledentistry and non-teledentistry encounters. Results cannot be tested for statistical significance, nor can they be generalized, but they can add to the literature for parties wishing to institute similar programs.

Previous research on adoption of teledentistry sheds similar light on the main challenges and best processes for development and implementation steps [28]. While policy and financing issues were not obstacles in our pilot, acceptance, perceived usefulness, and ease of use were. As our distribution of consults was not representative of the patient encounters across sites, staff at one pilot site may have been more enthusiastic about teledentistry, and interdisciplinary collaborations may benefit from teledentistry champions. With renewed engagement from valuable stakeholders as well as everyday users, additional and continued trainings regarding the intraoral camera and software, teledentistry could be considered more feasible and sustainable in this setting. As the study was conducted prior to the pandemic, similar programs may experience better utilization due to the current urgency and perhaps indefinite rationale behind providing remote care.

## Conclusions

Medical personnel largely used teledentistry for patients with dental trauma. Although utilization of teledentistry at medical sites was very low, caregivers, medical personnel, and dentists perceived benefits from using technology in this way. Consulting with a dentist via teledentistry may help medical personnel bridge knowledge and treatment gaps for patients who present to medical clinics with oral or dental concerns. Training medical personnel on using intraoral cameras and teledentistry software can alleviate issues during patient encounters and may increase uptake of new technology.

## Data Availability Statement

The raw data supporting the conclusions of this article will be made available by the authors, without undue reservation.

## Ethics Statement

The studies involving human participants were reviewed and approved by Nationwide Children's Hospital Institutional Review Board. Written informed consent from the participants' legal guardian/next of kin was not required to participate in this study in accordance with the national legislation and the institutional requirements.

## Author Contributions

All authors contributed to the design and implementation of the survey and research, to the analysis of the results, and to the writing of the manuscript.

## Funding

A Delta Dental Foundation Masters Thesis Award provided funding for printing of the surveys, patient and provider incentives to complete the surveys, and statistical consultation.

## Conflict of Interest

The authors declare that the research was conducted in the absence of any commercial or financial relationships that could be construed as a potential conflict of interest.

## Publisher's Note

All claims expressed in this article are solely those of the authors and do not necessarily represent those of their affiliated organizations, or those of the publisher, the editors and the reviewers. Any product that may be evaluated in this article, or claim that may be made by its manufacturer, is not guaranteed or endorsed by the publisher.
