# Supplementary material for: Connecting Medical Personnel to Dentists via Teledentistry in a Children's Hospital System: A Pilot Study
Source: Front Oral Health. 2021 Dec 9;2:769988. doi: 10.3389/froh.2021.769988 (PMC8757768; doi:10.3389/froh.2021.769988)
Supplement: Supplementary file 1 [file Data_Sheet_1.PDF]

Teledentistry- Dentist Survey

Patient's MRN (see email invitation): ______________________________

Your gender:
- ◯ male
- ◯ female
- ◯ other

Before this encounter, how many times have you used teledentistry in this setting?
- ◯ 0
- ◯ 1-5
- ◯ 6+

Using the videoconferencing software (Healthchat) was:
- ◯ Very Easy
- ◯ Easy
- ◯ Neutral
- ◯ Difficult
- ◯ Very Difficult

Directing and instructing medical staff to use the intraoral camera was:
- ◯ Very Easy
- ◯ Easy
- ◯ Neutral
- ◯ Difficult
- ◯ Very Difficult

	Strongly Agree	Agree	Neutral	Disagree	Strongly Disagree
The instructions for connecting and conducting a consult via Healthchat were clear and accurate.	◯	◯	◯	◯	◯
The intraoral camera gave me a view of the problem equivalent to if I were there in person.	◯	◯	◯	◯	◯
I could hear and visually assess the patient's area of concern as well as if I were there in person.	◯	◯	◯	◯	◯

	Strongly Agree	Agree	Neutral	Disagree	Strongly Disagree
I was comfortable with the teledentistry process.	◯	◯	◯	◯	◯
I was able to gather necessary information to make a recommendation or diagnosis I felt comfortable with, as I would with a non-teledentistry patient.	◯	◯	◯	◯	◯

There were technical problems during the visit that would not have been if I were there in person.	○	○	○	○	○

	Strongly Agree	Agree	Neutral	Disagree	Strongly Disagree
The patient was given the same recommendations as if I were there in person.	○	○	○	○	○
I think still photos rather than video would have allowed me to provide the same diagnosis.	○	○	○	○	○
I think the patient would have the same satisfaction if still photos rather than video were used.	○	○	○	○	○

	Strongly Agree	Agree	Neutral	Disagree	Strongly Disagree
I would prefer to consult patients similar to this patient via teledentistry rather than in person.	○	○	○	○	○
I would like this medical location to keep offering this teledentistry service to other patients.	○	○	○	○	○
I think that I would be able to treat more patients using teledentistry.	○	○	○	○	○

Comments:
Please offer any suggestions on how to improve the teledentistry experience for dentists, medical staff, or patients. ____________________________________
