# Supplementary material for: Connecting Medical Personnel to Dentists via Teledentistry in a Children's Hospital System: A Pilot Study
Source: Front Oral Health. 2021 Dec 9;2:769988. doi: 10.3389/froh.2021.769988 (PMC8757768; doi:10.3389/froh.2021.769988)
Supplement: Supplementary file 2 [file Data_Sheet_2.PDF]

Teledentistry- Medical Staff Survey

Patient's MRN (if you have it): __________________________________

Your demographics:
- ◯ RN
- ◯ LPN
- ◯ PCA
- ◯ Practitioner
- ◯ Other (explain)

____

Your age:
- ◯ under 30
- ◯ 31-40
- ◯ 41-50
- ◯ 51-60
- ◯ 61+

Your gender:
- ◯ male
- ◯ female
- ◯ other

	0	1-5	6+
Before this encounter, how many times have you used teledentistry in this setting?	◯	◯	◯
Before this encounter, how many times have you used telemedicine for other services?	◯	◯	◯

	Very Easy	Easy	Neutral	Difficult	Very Difficult
Using the videoconferencing software (Healthchat) was:	◯	◯	◯	◯	◯
Maneuvering the intraoral camera for live video was:	◯	◯	◯	◯	◯

The instructions for connecting to Healthchat were clear and accurate.
- ◯ Strongly Agree
- ◯ Agree
- ◯ Neutral
- ◯ Disagree
- ◯ Strongly Disagree

	Strongly Agree	Agree	Neutral	Disagree	Strongly Disagree
I was comfortable with the teledentistry process.	◯	◯	◯	◯	◯
I think the dentist understood the patient's problem.	◯	◯	◯	◯	◯

	Strongly Agree	Agree	Neutral	Disagree	Strongly Disagree
I think still photos rather than video would have allowed the dentist to provide the same diagnosis.	○	○	○	○	○

	Strongly Agree	Agree	Neutral	Disagree	Strongly Disagree
It takes more than one staff member to use the teledentistry equipment.	○	○	○	○	○
The teledentistry consult took an appropriate amount of time.	○	○	○	○	○
Talking with the dentist on the computer was a good use of the patient's time.	○	○	○	○	○
This consult method fits well into the 'process or flow' of this patient care site.	○	○	○	○	○

	Strongly Agree	Agree	Neutral	Disagree	Strongly Disagree
I would like this patient care site to keep offering this service to other patients.	○	○	○	○	○
Had the dentist not been available, the care of this patient would have been compromised.	○	○	○	○	○
I would recommend teledentistry to my colleagues.	○	○	○	○	○
I think that teledentistry improves the quality of services provided at this patient care site.	○	○	○	○	○
I like working and learning with the Dentistry department.	○	○	○	○	○

Comments:
Please offer any suggestions on how to improve the teledentistry experience for staff or patients ______________________________
