# Supplementary material for: Connecting Medical Personnel to Dentists via Teledentistry in a Children's Hospital System: A Pilot Study
Source: Front Oral Health. 2021 Dec 9;2:769988. doi: 10.3389/froh.2021.769988 (PMC8757768; doi:10.3389/froh.2021.769988)
Supplement: Supplementary file 3 [file Data_Sheet_3.PDF]

Teledentistry- Patient Survey

Patient's MRN (see email invitation): ______________________

Question 1. Someone explained to me that we were going to use teledentistry so that I knew what to expect

◯ Yes
◯ Maybe
◯ No

	Strongly Agree,	Agree,	Neutral,	Disagree,	Strongly Disagree
Question 2. I think the dentist could hear and see my child's problem as well as if the dentist were in the room.	◯	◯	◯	◯	◯
Question 3. I feel that my personal information was protected during the interaction with the dentist.	◯	◯	◯	◯	◯
Question 4. The dentist understood my child's problem.	◯	◯	◯	◯	◯
Question 5. The dentist responded to my concerns.	◯	◯	◯	◯	◯
Question 6. The dentist explained what's going on with my child's teeth or mouth.	◯	◯	◯	◯	◯
Question 7. I was comfortable with the teledentistry process.	◯	◯	◯	◯	◯
Question 8. If the dentist were here in person, there would not have been technical problems that interfered with the visit	◯	◯	◯	◯	◯
Question 9. My child was given the same recommendations as if the dentist were here in person.	◯	◯	◯	◯	◯
Question 10. The doctor here at this patient care site could have managed my child's needs fine without getting the dentist on the computer	◯	◯	◯	◯	◯
Question 11. I am satisfied with how long I got to talk to the dentist on the computer.	◯	◯	◯	◯	◯

Question 12. I am satisfied with how long I waited to talk to the dentist.	◯	◯	◯	◯	◯
Question 13. Talking with the dentist on the computer today was worth the time that I saved in not having to go to Nationwide Children's Main Emergency Department downtown.	◯	◯	◯	◯	◯

Question 14. In this location, I would prefer

◯ Dentist in-person,
◯ No preference,
◯ Dentist on the computer

Question 15. In this location, I would prefer

◯ Dentist on computer,
◯ No preference,
◯ No dentist available for today's visit

Question 16. In this location, I would prefer

◯ A dentist on computer,
◯ No preference,
◯ Go to NCH ED downtown to see in person

	Strongly Agree,	Agree,	Neutral,	Disagree,	Strongly Disagree
Question 17. I would like this patient care site to keep offering this service to patients.	◯	◯	◯	◯	◯
Question 18. Had I not had access to the dentist on the computer today, I would not know what to do for my child's problem	◯	◯	◯	◯	◯
Question 19. I would recommend the teledentistry process ot a friend in a situation similar to mine.	◯	◯	◯	◯	◯

Question 20. My insurance should cover this service.

◯ Yes
◯ Maybe,
◯ No

Comments:
Please offer any suggestions on how to improve the teledentistry experience for dentists, medical staff, or patients.

____
